# Exogenous BR delayed peach fruit softening by inhibiting pectin degradation enzyme genes

**DOI:** 10.3389/fpls.2023.1226921

**Published:** 2023-08-02

**Authors:** Jianzhao Li, Tingting Guo, Meiling Guo, Xiaonan Dai, Xiaofei Xu, Yanju Li, Zhizhong Song, Meixia Liang

**Affiliations:** ^1^ The Engineering Research Institute of Agriculture and Forestry, Ludong University, Yantai, Shandong, China; ^2^ College of Agriculture, Ludong University, Yantai, Shandong, China; ^3^ Yantai Academy of Agricultural Sciences, Yantai, Shandong, China

**Keywords:** peach, BR, pectin, *PpBES1*s, softening

## Abstract

Peach fruit deteriorates and senesces rapidly when stored at room temperature. Brassinosteroids (BRs) play an important role in regulating plant growth and development and maintaining fruit quality. However, little information is available on the effect of BRs on the senescence of harvested peach fruit. In this study, different concentrations of BR were used to treat ‘Hongniang’ peach fruit, and the results showed that 10 μM BR was the most beneficial concentration to delay the senescence of peach fruits. BR treatment delayed the decrease of fruit firmness, the release of ethylene, the increase in water-soluble pectin (WSP) and ionic-soluble pectin (ISP) content and the decrease in covalently bound pectin (CBP) content, inhibited the activities of pectin degradation enzymes, and inhibited the gene expression of *PpPME1/3, PpPG, PpARF2*, and *PpGAL2/16*. In addition, BR treatment also inhibited the expression of *PpBES1-5/6*. Cis-acting regulatory element analysis of pectin degradation enzyme promoters showed that many of them contained BES1 binding elements. All the above results showed that BR treatment had a positive effect on delaying the senescence of peach fruit and prolonging its storage period.

## 1 Introduction

Peach is a fruit native to China. It is loved by consumers all over the world because of its excellent flavor and rich nutrition. At present, China is the world’s peach production leader and has become a major producer (Cai et al., 2023). Peach fruits are typical respiratory mutant fruits (Kou et al., 2021). Improper storage after picking will lead to rapid softening of fruit hardness, loss of water and flavor, deterioration of taste, browning and decay within the fruit, and sharp decline in fruit quality, which will eventually lead to shortened postharvest life and loss of commercial value (Barry and Giovannoni, 2007; Tucker et al., 2017). Usually, the most common means of fruit storage is low temperature (Zhang et al., 2011). However, if the low temperature storage time is too long, it will lead to chilling injury of peach fruit, so it is particularly important to choose a suitable storage method (Wang et al., 2019; Song et al., 2022; Zhao et al., 2021).

The cell wall acts as a barrier to fruits, and the integrity of the cell wall is inseparable from fruit senescence and softening. The decrease in pectin content in the fruit cell wall was related with cell wall degradation enzyme activity, and the genes expression of cell wall modification enzymes (Marín-Rodríguez et al., 2002; Shi et al., 2022). Pectin is an important component of the plant cell wall. Studies have shown that water-soluble pectin (WSP), ionic-soluble pectin (ISP), and covalently bound pectin (CBP) play an important role in maintaining cell wall structural integrity (Kozioł et al., 2017). In general, the pectin in cell wall of fruits is degraded in the later stage of ripening. The metabolism of cell wall components results in a decrease in fruit firmness (Liu et al., 2017). The change in fruit texture is closely related to cell wall pectin-degrading enzymes, and the main functional enzymes are polygalacturonase (PG), pectin lyase (PL), pectin methylesterase (PME), arabinofuranosidase (α-ARF), and β-galactosidase (β-GAL). Reducing the activity of these enzymes can greatly improve the firmness of fruits, and delay the senescence of postharvest fruits (Micheli, 2001; Brummell, 2006; Chang et al., 2017; Huang et al., 2022).

Brassinosteroid (BR) is a kind of polyhydroxylated steroid phytohormone that is recognized as the sixth largest phytohormone and has been widely used in horticulture (Ali, 2017). BR plays an important role in regulating plant growth and development, maintaining fruit quality, increasing fruit yield and reducing postharvest losses (Kou et al., 2021). Studies have shown that the application of BR in carambola fruit can enhance antioxidant capacity and improve respiratory pathways to delay the senescence of carambola and improve fruit quality (Zhu et al., 2021). Brassinosteroids effectively inhibited the development of blue mold in jujube fruit, enhanced the activity of defense-related enzymes, and significantly delayed fruit senescence by reducing ethylene production and maintaining fruit quality (Zhu et al., 2010). In addition, BR treatment increased the activity of antioxidant enzymes and the content of single phenolic compounds in carambola fruit, inhibited membrane lipid peroxidation, maintained the integrity of the cell membrane, and ensured the quality of fruit (Duan et al., 2022). Strawberry fruit treated with 1 μM EBL showed lower decay extension, microbial quantity, weight loss and postharvest life during storage and enhanced the potential of strawberry fruit nutrition and overall quality, plant compounds and postharvest life (Sun et al., 2020). EBR delayed changes in the respiration rate, flesh color and ascorbic acid content of kiwifruit, inhibited the accumulation of superoxide anions and hydrogen peroxide in kiwifruit, and delayed the ripening and senescence of kiwifruit (Lu et al., 2019; Wang et al., 2020).

BES1/BZR1 is a transcriptional regulator that regulates the expression of related genes in BR downstream signaling pathways. It also plays a vital role in BR signal transduction, and plays an important regulatory role in plant growth and development events regulated by various signals through direct interaction with other key proteins or genes (Kim et al., 2009; Li et al., 2018). Liu et al. (2021) found that SlBES1 promotes tomato fruit softening by transcriptionally inhibiting *PMEU1*. In Arabidopsis, BZR1 is involved in plant cold tolerance by regulating the expression of *AtPME41* (Qu et al., 2011). MaBZR1/2 in banana interacts with MaMPK14 to enhance the transcriptional repression of cell wall modification genes (including *MaEXP2*, *MaPL2* and *MaXET5*) to promote banana ripening (Shan et al., 2020).

BR has been widely used to improve the postharvest quality of fruits, but there are few studies on BR in postharvest storage of peach fruits. The purpose of this study was to investigate the effects of exogenous BR on delaying postharvest senescence and fruit quality attributes of peach fruit and its mechanism from the changes of fruit hardness, ethylene release, soluble solids, titratable acid, pectin content, pectin degradation enzyme activity, pectin degradation gene expression, and brassinosteroid-related transcription factor expression.

## 2 Materials and methods

### 2.1 Fruit materials

In this study, we used ‘Hongniang’ peach fruits as experimental material to pick peach fruits from Xieziya Village (longitude: 37.4381, latitude: 121.1726) in Fushan District, Yantai City, Shandong Province, on September 27, 2022. Each peach fruit sample used in our experiments was carefully selected, all fruits were free of pests and diseases and had obvious mechanical damage, and the maturity and size of the fruits were basically the same. After sampling, the fruits were immediately transported back to the laboratory for processing, and then 15 peach fruits were selected for sampling and used as 0 d for all treatments in this experiment. The remaining fruits were divided into an experimental group and a control group.

### 2.2 Postharvest treatment

10 μM, 20 μM, and 30 μM BR-treated peaches were set as the experimental group, deionized water-treated peach fruits were set as the control group, and three biological replicates were set up for each treatment. After treatment, samples were taken every four days, peeled, chopped, and quick-frozen with liquid nitrogen before being placed in a -80°C freezer for subsequent determination of cell wall pectin content, cell wall-degrading enzyme activity, and RNA extraction.

### 2.3 Determination of fruit firmness, ethylene content, soluble solids content and fruit acid content

The firmness of each fruit was determined using a GY-4 texture analyzer with a 7.9 mm diameter probe (Zhejiang Top Instruments Co., Ltd., Hangzhou, China). Two firmness measurement points for each fruit were measured in the middle of both sides of the symmetrical suture of each fruit. The puncture depth was 10 mm, and the firmness unit was Newtons (N). Ethylene content was detected using a Shimadzu meteorological chromatograph (GC-2014) with three ethylene biological replicates per treatment set. The soluble solids content and fruit acidity were measured using a PAL-BX/AACID 14 pocket refractometer (Japanese ATAGO).

### 2.4 Extraction and determination of pectin and pectin degradation enzyme activity

The extraction of pectin from peach fruits was modified according to the method described by Li et al. (2023). The determination of pectin was modified according to the method described by Jia et al. (2019). We detected polygalacturonase (PG) enzyme activity according to the method of Cao et al. (2014) with slight modifications. Pectin methylesterase (PME) enzyme activity was measured according to Basak and Ramaswamy (1996). The determination of pectate lyase (PL) activity was slightly adjusted according to the method of Payasi and Sanwal (2003).

### 2.5 RNA extraction and qRT-PCR-based analysis

The FastPure Plant Total RNA Isolation Kit (Vazyme, Nanjing, China) was used to extract total RNA from fruits, and HiScript III-RT SuperMix (Vazyme, Nanjing, China) was used to synthesize cDNA. We used the ChamQ Universal SYBR qPCR Master Mix Kit (Vazyme, Nanjing, China) to conduct real-time PCR on a Bio-Rad CFX96 real-time system (Bio-Rad, CA, USA).

Gene-specific primers were designed by using primer 3 (http://bioinfo.ut.ee/primer3-0.4.0) (**SupplementaryTable 1**). The primer efficiency was tested by using a standard curve. Primers with efficiencies between 80% and 120% were used for qRT-PCR. The relative quantification method (2^-ΔΔCt^) was used by comparison with the PpActin gene (Wang et al., 2018). Three biological replicates for each sample were conducted.

### 2.6 Statistical analysis

The experimental data were analyzed by using SPSS 19.0 software. Student’s t test (P <0.05) was used to calculate pairwise comparisons. Statistically significant differences (P <0.05) are represented by stars, and these values are expressed as the average of three biological replicates. GraphPad Prism 6 software (La Jolla California, USA) was used for graphing.

## 3 Results

### 3.1 Effect of BR on the quality of ‘Hongniang’ peach fruit

**Figure 1:**
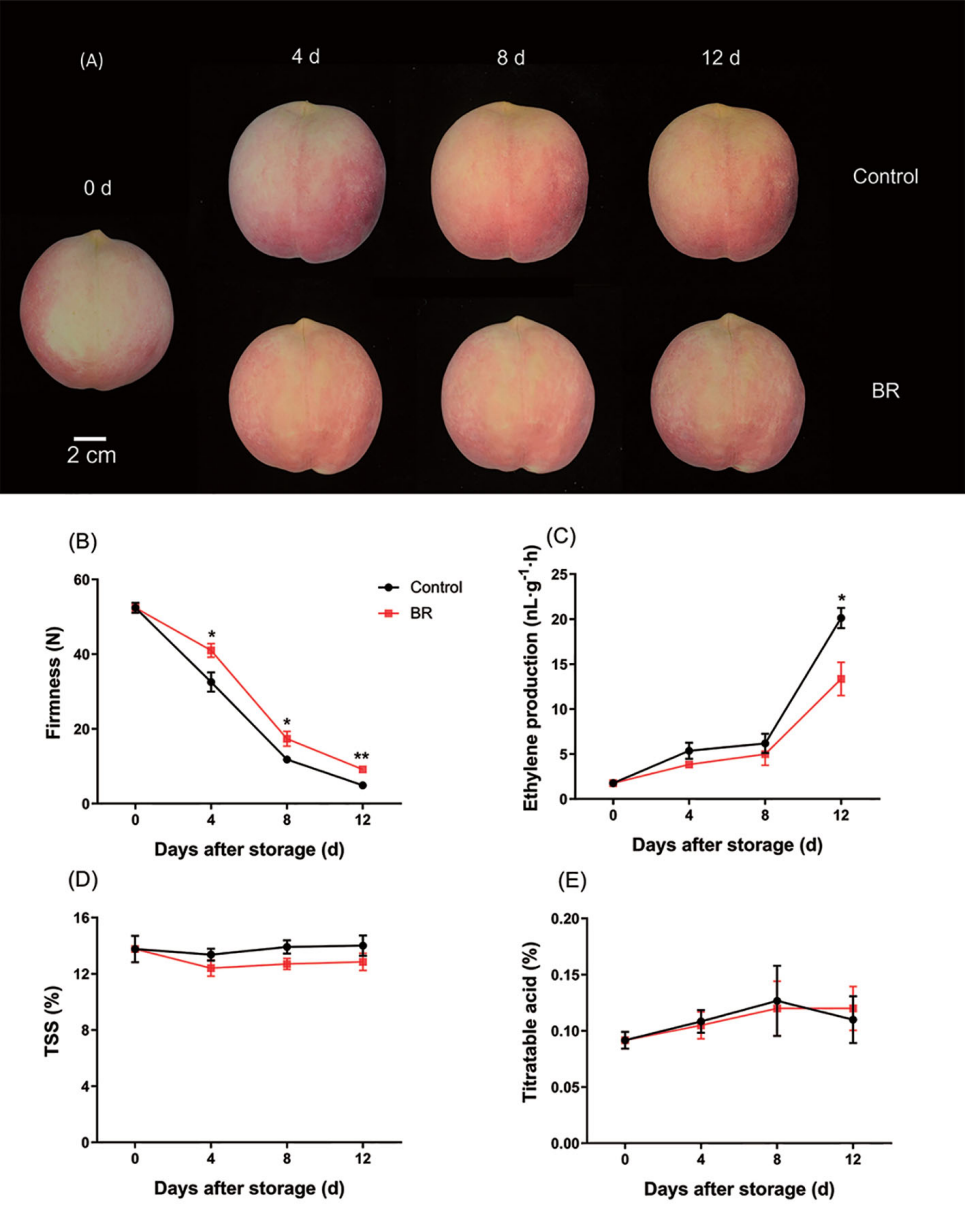
Effect of BR treatment on the appearance and physiological indices of ‘Hongniang’ peaches. **(A)** The appearance of ‘Hongniang’ peaches during 12 d of storage. **(B)** Changes in firmness of ‘Hongniang’ peach fruit under control and BR treatment. **(C)** Changes in ethylene production content of peach fruit under control and BR treatment. **(D)** Changes in total soluble solids (TSS) content in peach fruit under control and BR treatment. **(E)** Changes in titratable acid (Acid) content in peach fruit under control and BR treatment. Error bars represent the SE of the three replicates. *, and ** indicate significant differences of *p <*0.05 and *p <*0.01, respectively.

We treated ‘Hongniang’ peach fruit with 10 μM, 20 μM, and 30 μM BR and used deionized water-treated peach fruit as the control group, and it was found that the 10 μM BR-treated peach fruit maintained the firmness best (**Supplementary Figure 1**). Then, we selected 10 μM BR-treated peach fruit samples for subsequent research. There were no particularly significant differences in fruit appearance during storage between the BR-treated group and the control group (**Figure 1A**). The firmness of peach fruits gradually decreased during the shelf life, but BR effectively delayed this process. The firmness of the freshly picked peach fruits was approximately 52 N, and the firmness of the control group after storage for 12 days was approximately 4.9 N, the firmness of the BR-treated peach fruits was approximately 9 N, and the firmness of the BR-treated fruit was approximately 2 times that of the control group (**Figure 1B**). Ethylene production rate of BR-treated fruits and the control group showed a slow upward trend from 0 d to 8 d, the ethylene yield of the control group increased sharply from 8 d to 12 d, and the ethylene yield increased from approximately 6 nL·g^-1^·h to 20 nL·g^-1^·h. The same trend was followed in the experimental group, but the ethylene yield in the experimental group at 12 d was approximately 13 nL·g^-1^·h (**Figure 1C**). There were no significant differences in TSS and acid content between BR-treated and control peach fruit (**Figures 1D, E**).

### 3.2 Effects of BR treatment on the content of different types of pectin in ‘Hongniang’ peach fruit

**Figure 2:**
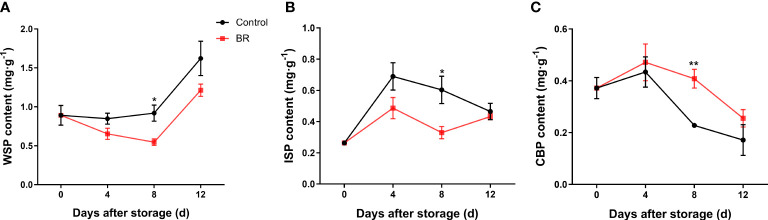
Effects of BR treatment on the content of different types of pectin in the fruit of ‘Hongniang’ peaches. **(A)** The content of water-soluble pectin (WSP) under the control and BR treatments. **(B)** The content of ionic-soluble pectin (ISP) under control and BR treatment. **(C)** The content of covalently bound pectin (CBP) under control and BR treatment. Error bars represent the SE of the three replicates. *, and ** indicate significant differences of *p <*0.05 and *p <*0.01, respectively.

During the whole storage period, with the softening of the fruit, the content of water-soluble pectin (WSP) in the BR-treated group and the control group increased continuously, but BR treatment prevented the increase in WSP content (**Figure 2A**). At 12 days of storage, the contents of WSP in the two groups were approximately 1.62 mg · g^-1^ and 1.21 mg · g^-1^, respectively, and the BR-treated group was significantly lower than the control group (**Figure 2A**). The content of ionic-soluble pectin (ISP) in the control group was significantly higher than that in the BR-treated group (**Figure 2B**). The content of covalently bound pectin (CBP) was negatively correlated with the ripeness of fruits. With the continuous extension of the storage period and the continuous ripening and softening of peach fruits, the content of CBP in the fruits continued to decrease, and the content of CBP in the control group decreased significantly after 4 d of storage, while BR treatment effectively alleviated this phenomenon (**Figure 2C**).

### 3.3 Effect of BRs on pectin-degrading enzyme activity in ‘Hongniang’ peaches

**Figure 3:**
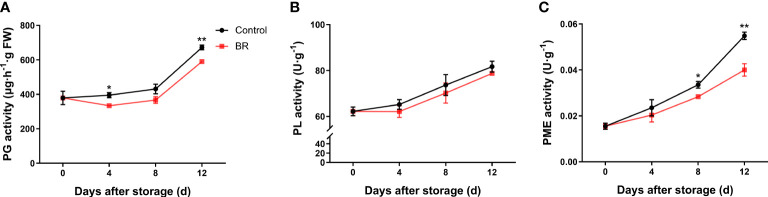
Effect of BR treatment on the activities of different types of pectin degradation enzymes. **(A)** Polygalacturonase (PG) activity under control and BR treatment. **(B)** Pectin lyase (PL) activity under control and BR treatment. **(C)** Pectin methylesterase (PME) activity under control and BR treatment. Error bars represent the SE of the three replicates. *, and ** indicate significant differences of *p <*0.05 and *p <*0.01, respectively.

In both the BR and control groups, the activity of polygalaccuronidase (PG) showed an upward trend (**Figure 3A**). At the end of the shelf life, the PG activity was 590 μg/h·g FW in the BR-treated peach fruit and approximately 672 μg/h·g FW in the control group. The pectin lyase (PL) activity of fruits in the experimental and control groups was approximately 78 U·g^-1^ and 81 U·g^-1^ after 12 days of storage, respectively (**Figure 3B**). The activity of pectin methylesterase (PME) in the control group increased from 0.033 U·g^-1^ to 0.054 U·g^-1^ at 8 to 12 days of storage, while the activity of PME in BR-treated fruit increased from 0.028 U·g^-1^ to 0.040 U·g^-1^ after 8 to 12 days of storage (**Figure 3C**). The above results showed that BR treatment could effectively inhibit the activity of three pectin degradation enzymes.

### 3.4 Correlation analysis between firmness, ethylene, TSS, acid, WSP, ISP, CBP, PG, PL and PME of ‘Hongniang’ peach fruit

To determine whether the softening of the fruit is affected by the above factors, we analyzed whether there is a correlation among them (**Table 1**). Except for the positive correlation between CBP and fruit firmness, the other indicators were negatively correlated with fruit firmness. In particular, the activity of PME was significantly negatively correlated with firmness, and the correlation coefficient was -0.87. Ethylene production was significantly positively correlated with WSP content and the activities of PG, PL and PME, with correlation coefficients of 0.82, 0.90, 0.80 and 0.91, respectively. There was a positive relationship among WSP and PG, PL, and PME activity. CBP was negatively correlated with PG, PL and PME. There was a positive relationship among the activities of the three pectin degradation enzymes.

**Table 1:** Correlation analysis among firmness, ethylene, TSS, acid, WSP, ISP, CBP, PG, PL and PME.

	Firmness	Ethylene	TSS	Acid	WSP	ISP	CBP	PG	PL	PME
Firmness										
Ethylene	-0.73***									
TSS	-0.04	0.17								
Acid	-0.23	0.14	-0.21							
WSP	-0.47*	0.82***	0.14	0.22						
ISP	-0.21	0.08	0.13	0.16	0.01					
CBP	0.67***	-0.61**	-0.39	0.04	-0.51*	-0.04				
PG	-0.68***	0.90***	0.25	0.05	0.86***	0.01	-0.69***			
PL	-0.76***	0.80***	0.03	0.28	0.66**	-0.07	-0.58**	0.81***		
PME	-0.87***	0.91***	0.09	0.11	0.72***	0.11	-0.69***	0.87***	0.85***	

*, **, and ***indicate significant differences of p <0.05, p <0.01, and p <0.001, respectively.

### 3.5 Effect of BRs on the expression of pectin degradation enzyme genes in ‘Hongniang’ peaches

**Figure 4:**
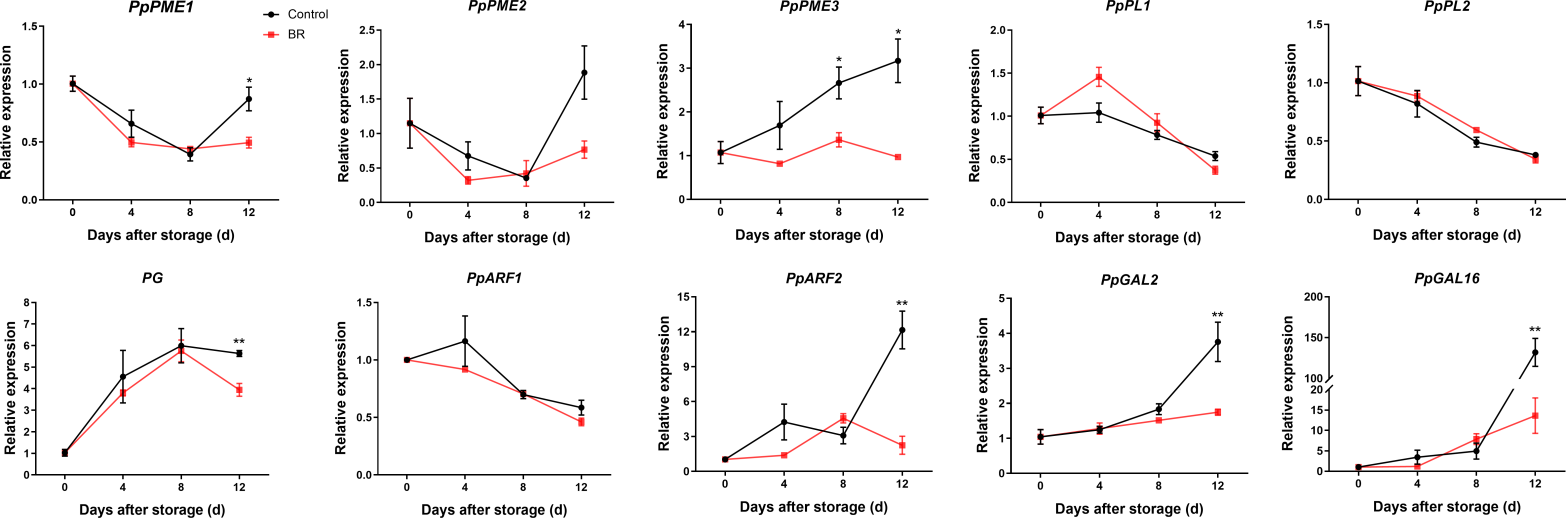
Relative expression levels of pectin degradation enzyme genes in control and BR-treated ‘Hongniang’ peach fruit. Error bars represent the SE of three replicates. *, and ** indicate significant differences of *p <*0.05 and *p <*0.01, respectively.

To further uncover the causes of softening of the ‘Hongniang’ peach fruit, we selected several of the more common pectinase genes that degrade pectin, including PME, PL, PG, Arabinofuranosidase (ARF), and β-galactosidase (GAL), and analyzed their relative expression levels in BR-treated and control gruops (**Figure 4**). The expression levels of *PpPME1/2* in the BR treatment and control groups were basically consistent; the gene expression of the control group was higher than that of the experimental group at 4 d and basically the same as that of the experimental group at 8 d, and the gene expression of the control group showed a rapid upward trend from 8 to 12 d, while BR treatment significantly alleviated this trend. The expression level of *PpPME3* was inhibited by BR, and the gene expression of the control group was higher than that of the BR-treated group at 4 d to 12 d, and the gene expression of the control group was approximately 3 times that of the experimental group at 12 d. The gene expression level of *PpPL1/2* was basically the same in the control group, and the expression level of the gene gradually decreased with the extension of shelf life. The gene expression trend of *PpPG* in the BR and control groups was almost identical, and the gene expression of *PpPG* in both groups continued to increase at 0 d to 8 d and decreased at 12 d. The expression trend of the *PpARF1* gene in both the BR group and the control group slowly declined. The gene expression level of *PpARF2* in the BR group and the control group had the opposite trend with increasing storage time, and the expression level of the two was quite different at 12 days. The expression trend of *PpGAL2/16* was consistent in the BR group and the control group, and the expression gradually increased during the extension of the storage period. Notably, in the late stage of fruit storage, there was a significant difference in the expression of *PpGAL2/16* between the BR group and the control group. The gene expression of *PpGAL16* in the control group was approximately 131.8 at 12 days, while the expression of the BR group was only approximately 13.6.

### 3.6 Effects of BR treatment on PpBES1 gene expression

**Figure 5:**
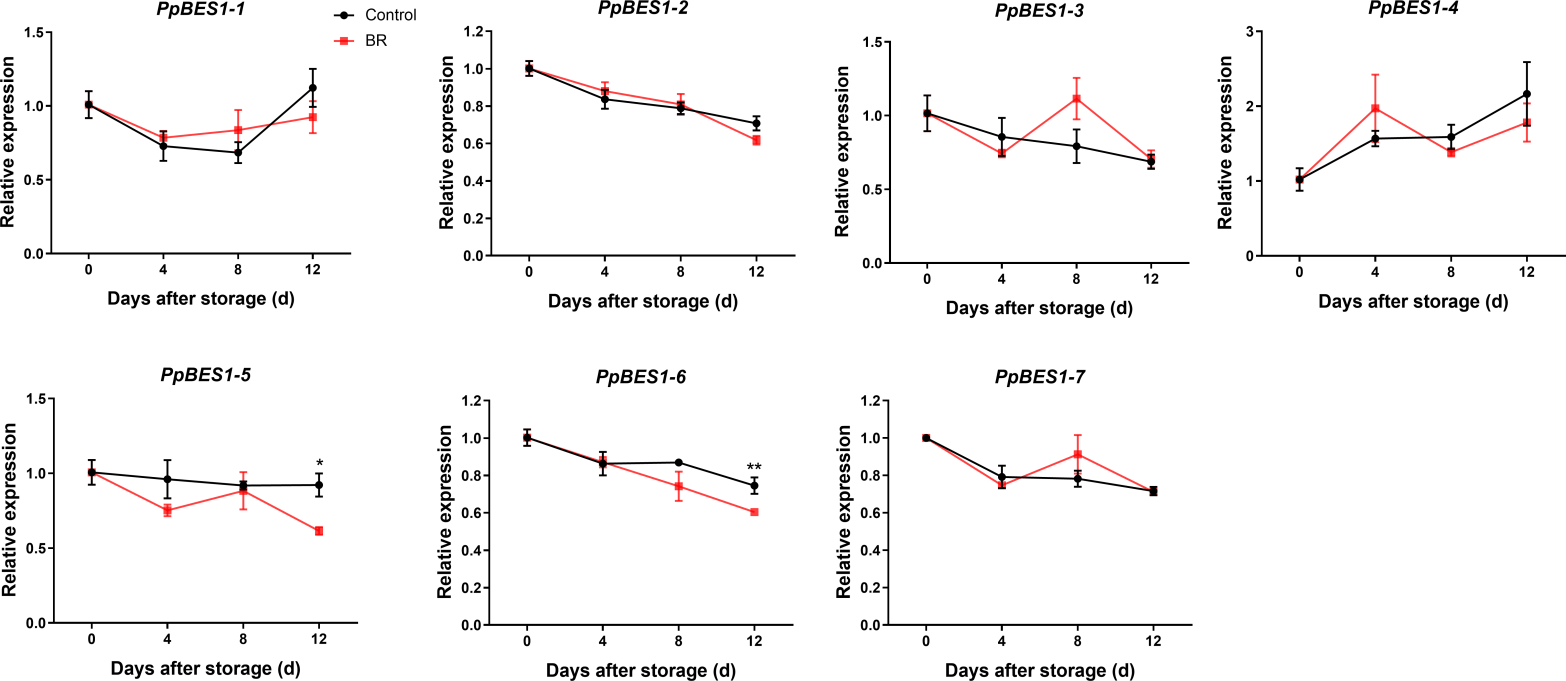
Relative expression levels of the *PpBES1*s gene under control and BR treatment. Error bars represent the SE of the three replicates. *, and ** indicate significant differences of *p <*0.05 and *p <*0.01, respectively.

To analyze BR signal transduction in peach fruit, we checked the expression of the BR-responsive transcription factors *PpBES1*s in the BR and control groups. It is worth noting that the gene expression of *PpBES1-5/6* in the BR group and the control group was significantly different, and the gene expression of *PpBES1-5/6* in the BR group was inhibited compared with that in the control group (**Figure 5**). During the whole storage period of peach fruit, there was no significant difference in the expression of *PpBES1*s genes between the control group and the treatment group.

### 3.7 Correlation of *PpBES1*s with pectin-degradation enzyme genes

**Figure 6:**
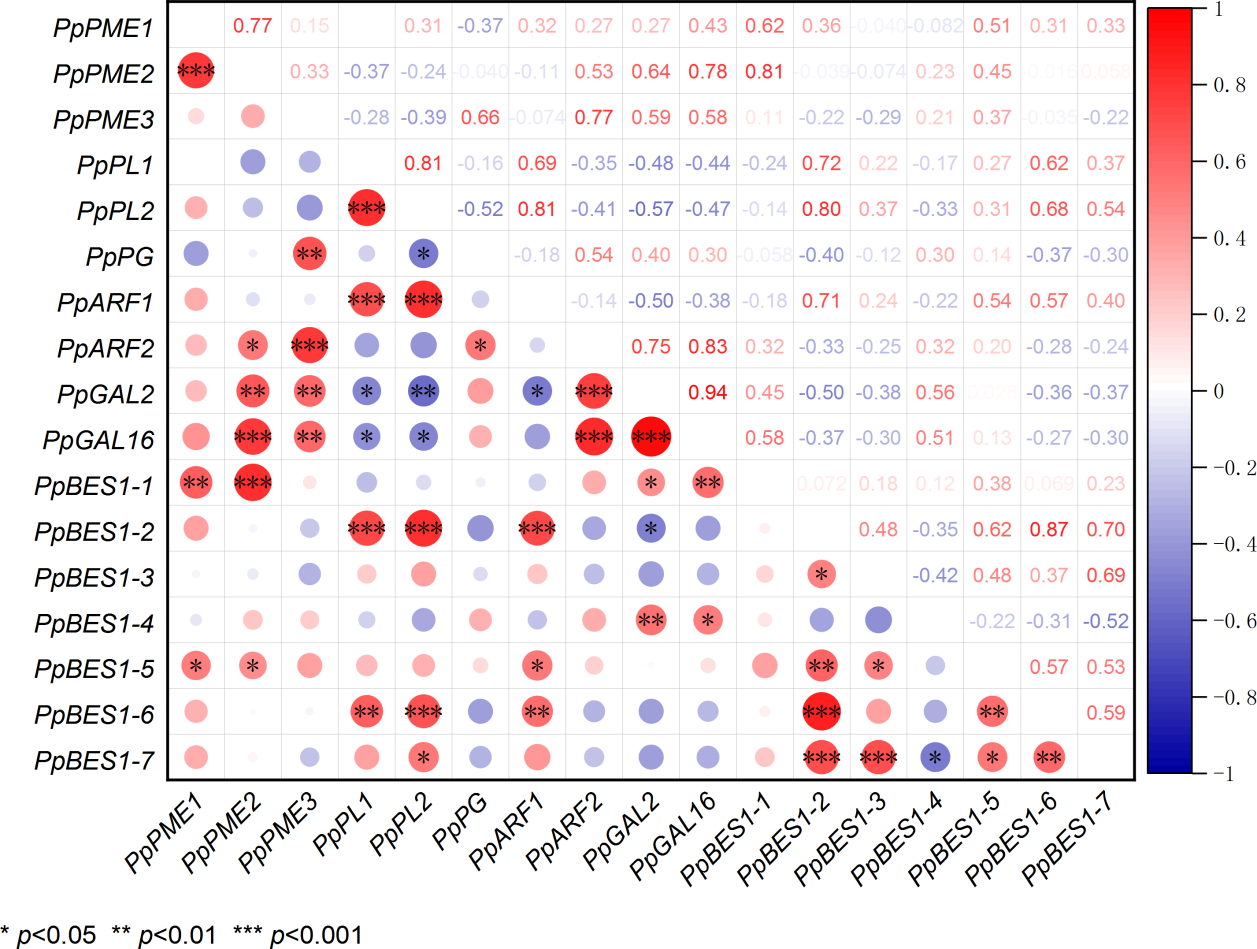
Correlation analysis of *PpBES1*s and pectin degradation enzyme genes. Red dots indicate positive correlations, and blue dots represent negative correlations. The size of the circles indicates the degree of the intergene correlation. *, **, and ***indicate significant differences of *p <*0.05, *p <*0.01, and *p <*0.001, respectively.

To determine whether there is a relationship between *PpBES1*s and pectin degradation enzyme genes, we performed correlation analysis using their gene expression levels. As shown in **Figure 6**, there was a significant positive correlation between *PpBES1-1* and *PpPME1*, *PpPME2*, *PpGAL2*, and *PpGAL16* (**Figure 6**). There was a significant positive correlation between *PpBES1-2* and *PpPL1*, *PpPL2*, and *PpARF1*. There was a significant negative correlation between *PpBES1-2* and *PpGAL2*. There was a significant positive correlation between *PpBES1-4* and *PpGAL2* and *PpGAL16*. There was a significant positive correlation between *PpBES1-5* and *PpPME1*, *PpPME2*, and *PpARF1*. There was a significant positive correlation among *PpBES1-6* and *PpPL1*, *PpPL2*, and *PpARF1*. There was a significant positive correlation between *PpBES1-7* and *PpPL2*. Finally, we analyzed the cis-acting elements of all pectin degrading enzyme genes and found that most promoters of these genes contained BES1 protein binding sites (E-box, BRRE) (**Figure S2**).

## 4 Discussion

### 4.1 Senescence of peach fruits

Fruits with more respiration release a large amount of ethylene during ripening, and inhibiting the production of ethylene is one of the methods for postharvest fruit storage and preservation (Wang et al., 2020). Firmness and sugar content are important parameters to measure changes during fruit storage. We found that BR treatment significantly inhibited ethylene release compared with the control group (**Figure 1B**). BR treatment also suppressed pear and apple ethylene production (Ji et al., 2021). In peach fruits, the quality of flavor depends to some extent on soluble solids, such as sugar and organic acids, which determine the sweetness and sourness of peach fruits (Hu et al., 2020). Sugar is one of the main factors affecting the edible quality of fruits. The high maturity of fruits may be accompanied by a decrease in firmness. Moreover, fruit firmness and sugar content are also some of the main measures that affect consumers’ satisfaction with peach fruits (Cirilli et al., 2016). The results of this experiment showed that treatment with 10 μM BR could effectively maintain the hardness and sugar content of the fruits at a certain level, which did not affect the taste and was conducive to storage (**Figures 1C, D**), which was consistent with the results of Lu et al. (2019).

### 4.2 Effects of BR treatment on cell wall substances, enzyme activity and gene expression

The decrease in fruit firmness during fruit ripening is largely due to the disintegration of the cell wall, which reduces the rigidity of the cell wall and the adhesion between cells (Li et al., 2010). The degradation of polysaccharides and changes in the bonding between polymers lead to increased cell separation and wall softening and swelling, combined with changes in swelling pressure, resulting in fruit softening and structural changes (Peng et al., 2022). Usually, the ripening and senescence of fruits are accompanied by an increase in the content of water-soluble pectin (WSP) and ionic-soluble pectin (ISP) and a decrease in the content of covalently bound pectin (CBP) (Chen et al., 2015). We found that the fruits treated with BR inhibited the increase in water-soluble pectin and ionic-soluble pectin content and the decrease in covalently bound pectin content (**Figures 2A, B, C**).

Studies have shown that the decomposition of pectin and the destruction of the cell wall structure are usually caused by increases in PG, PL and PME enzyme activities (Chen et al., 2021). Among them, PG is the most studied cell wall degradation enzyme and plays a key role in fruit softening (Dautt-Castro et al., 2019). PME methylates pectin and destroys the integrity of the cell wall (Xu et al., 2022). In addition, 1-MCP reduced the activities of polygalacturonase (PG), pectin methylase (PME) and pectin lyase (PL) by regulating auxin signal transduction, and down-regulated the expression of *PpPG1*, *PpPG2*, *PpPME1*, *PpPME2*, *PpPME1* and *PpPEL2*, which alleviated the cell wall degradation of peach fruit (Zheng et al., 2023). In our results, we found that BR inhibited the enzyme activity of PG, PL, and PME (**Figure 3**). There is sufficient evidence that the activities of PG and PME are significantly negatively correlated with fruit firmness (Ge et al., 2020). In the fruits treated with BR, fruit hardness was negatively correlated with the activity of PG, PL, and PME (**Table 1**). Tatsuki et al. (2021) identified that low temperature delays the decrease in fruit firmness by inhibiting the expression of the cell wall-modifying enzyme gene *PpPGM*. Xu et al. (2022) found that gene silencing of *PpePL1* and *PpePL15* effectively delayed the decrease of peach fruit hardness and also reduced the degradation of cell wall. In apple, *MdPG1* expression is ethylene-dependent and involved in apple fruit ripening (Costa et al., 2010). In mango fruits, the expression level of *MaPG*s increased with the advancement of maturity (Dautt-Castro et al., 2019). In our results, we found that the expression levels of *PpPME3*, *PpPG*, *PpARF2*, *PpGAL2* and *PpGAL16* were inhibited by exogenous BRs compared with the control group (**Figure 4**).

### 4.3 Effects of BR treatment on the expression of *PpBES1*s

As a key component of BR signaling pathway, BZR transcription factor may play a role in fruit ripening through transcriptional regulation. BES1 can directly bind to gene promoters to regulate their expression. BZR1/BES1 usually binds to E-box to activate gene expression and binds to BRRE to repress gene expression (Nolan et al., 2020). PuBZR1 inhibits the expression of the transcription factor *PuERF2* by binding to its promoter. PuERF2 binds to the promoters of *PuACO1* and *PuACS1a*, and BR-activated BZR1 inhibits ACO1 activity and the expression of *ACO1* and *ACS1*, thereby reducing ethylene production and inhibiting pear and apple fruit ripening (Ji et al., 2021). In our research results, it was found that the expression of some *PpBES1s* was inhibited after the application of BR, especially in the later stage of storage (**Figure 5**). Similarly, the transcription level of DkBZR1 decreased slightly during persimmon fruit ripening after EBR application (He et al., 2021). In addition, Guo et al. (2019) also found that BR treatment inhibited the expression levels of *MaBZR1/2.* Our correlation analysis also showed that the expression of pectin degradation enzyme genes were positively correlated with the expression of *PpBES1*s (**Figure 6**). Subsequently, we analyzed the promoters of pectin degradation enzyme genes and found that there were many E-box (CANNTG) and BRRE (CTGTC/CG) motifs, which BES1 can directly bind (**Figure S2**). All these results suggested that BR might regulate the expression of pectin degradation genes by inhibiting the transcription of *PpBES1*s. The molecular mechanisms of how PpBES1s regulate the expression of pectin degradation enzyme genes need to be further studied.

## 5 Conclusions

This study reported the positive effect of BR on delaying peach fruit softening during postharvest storage. BR treatment significantly reduced the decrease in fruit firmness and the increase in ethylene production without affecting the commercial quality of fruits. In addition, BR treatment inhibited the contents of WSP and ISP and maintained the content of CBP. BR also inhibited the activity of pectin degradation enzymes and gene expression of *PpPME3*, *PpPG*, *PpARF2*, *PpGAL2*/*16*, and *PpBES1*-*5*/*6*. Correlation analysis and promoter analysis suggested that BR inhibited the expression of pectin degradation genes by inhibiting the transcription of *PpBES1*s. In summary, all the physiological, biochemical and molecular evidence showed that BR treatment enhanced the storage of peach fruits.

## Data availability statement

The original contributions presented in the study are included in the article/**Supplementary Material**. Further inquiries can be directed to the corresponding author.

## Author contributions

JL and ML conceived and supervised the project. JL and ML designed the experiments. JL and TG performed most of the experiments. MG, XD, XX, YL, and ZS carried out some of the experiments. TG and JL analyzed the data and wrote the manuscript. All authors contributed to the article and approved the submitted version.
