# Supplementary material for: Exogenous BR delayed peach fruit softening by inhibiting pectin degradation enzyme genes
Source: Front Plant Sci. 2023 Aug 2;14:1226921. doi: 10.3389/fpls.2023.1226921 (PMC10436216; doi:10.3389/fpls.2023.1226921)
Supplement: Supplementary file 1 [file DataSheet_1.docx]

Table S1 Primer sequences used in this study.

| Gene Name | Primer Sequence (5’-3’) |
| --- | --- |
| *PpACTIN*-F | TAATGCGGGAAAGATACTGGA |
| *PpACTIN*-R | AGGAGGACGCACAACCACAT |
| *PpBES1-1*-F | TGAAGCCTGTTGTTGCTCAC |
| *PpBES1-1*-R | AAGGGTTGTGGCAGATGAAC |
| *PpBES1-2*-F | GGGTGGCTGAAATTGGTAGA |
| *PpBES1-2*-R | ACGGAAGCTTCTCATGCAGT |
| *PpBES1-3*-F | TTACCGAAAGGGATGCAAAC |
| *PpBES1-3*-R | TTGGGGAAGCTTGGATGTAG |
| *PpBES1-4*-F | TGCATTTTCTCACCAACCAA |
| *PpBES1-4*-R | GAGGCTGTGGTCGAACTCTC |
| *PpBES1-5*-F | TCCATTAAGCTCCCCAACTG |
| *PpBES1-5*-R | ATGGGTTCAAAGCAACAAGG |
| *PpBES1-6*-F | TAGCCTGGGATCAAGGATTG |
| *PpBES1-6*-R | GCAAATTGCCCCTTGAACTA |
| *PpBES1-7*-F | CTCAGCTTTCCCAAGTCCTG |
| *PpBES1-7*-R | AAGGGAGTCCCAGTCAGGTT |
| *PpPME1*-F | AGATCATTCTGCAAGTCCAC |
| *PpPME1*-R | TATCGCAACTTGGAGAGACT |
| *PpPME2*-F | TGAACTGGTCTCTCTCTGCT |
| *PpPME2*-R | TACTGCATGTGTCCTGGTTA |
| *PpPME3*-F | CGCGACAAGACTGTGTATTA |
| *PpPME3*-R | CCCACTTTGTTCCTCTGATA |
| *PpPL1*-F | CAGCAAAACCCTGAATTGGT |
| *PpPL1*-R | TGAATCCGTGACCACGTAAA |
| *PpPL2*-F | TTGCCATGGTTGACATGAGT |
| *PpPL2*-R | GGGTCTGGGGTTAACAGGAT |
| *PpPG*-F | TCTGCATGGGCTAAAGCTTGT |
| *PpPG*-R | TGCGGGCACATAAATGACA |
| *PpARF1*-F | GGGCTGAGCTTGTAAGCAAC |
| *PpARF1*-R | AGCTACTGGCACCTTGGCTA |
| *PpXYL*-F | GCTGCTGGTGAACAATGCTA |
| *PpXYL*-R | GTACATTGCCCTTGCCTCAT |
| *PpGAL2*-F | GCATTACAACCTGCCTCCAT |
| *PpGAL2*-R | CCGGCTGTGGTAAATGAAGT |
| *PpGAL16*-F | TTGCGCTGTTTATTGCAAAG |
| *PpGAL16*-R | CTGCAGCATGCAATTCCTTA |


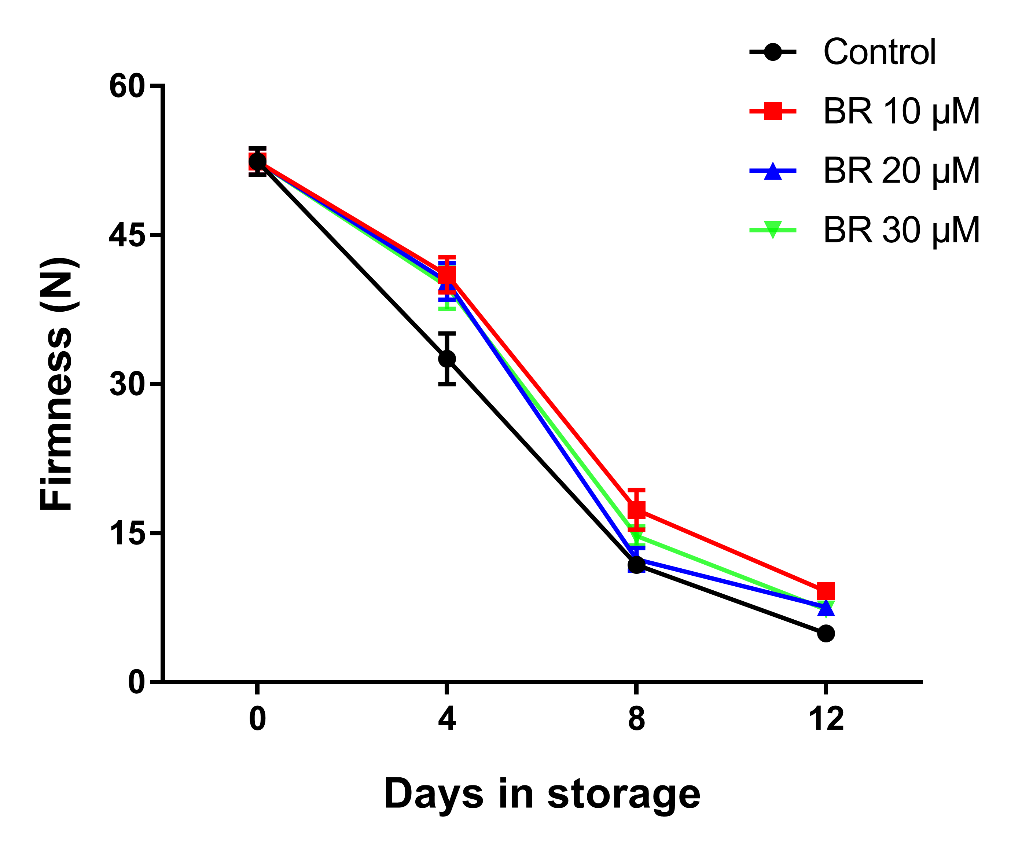


Figure. 1 The firmness of 'Hongniang' peaches treated with three concentrations of BR.


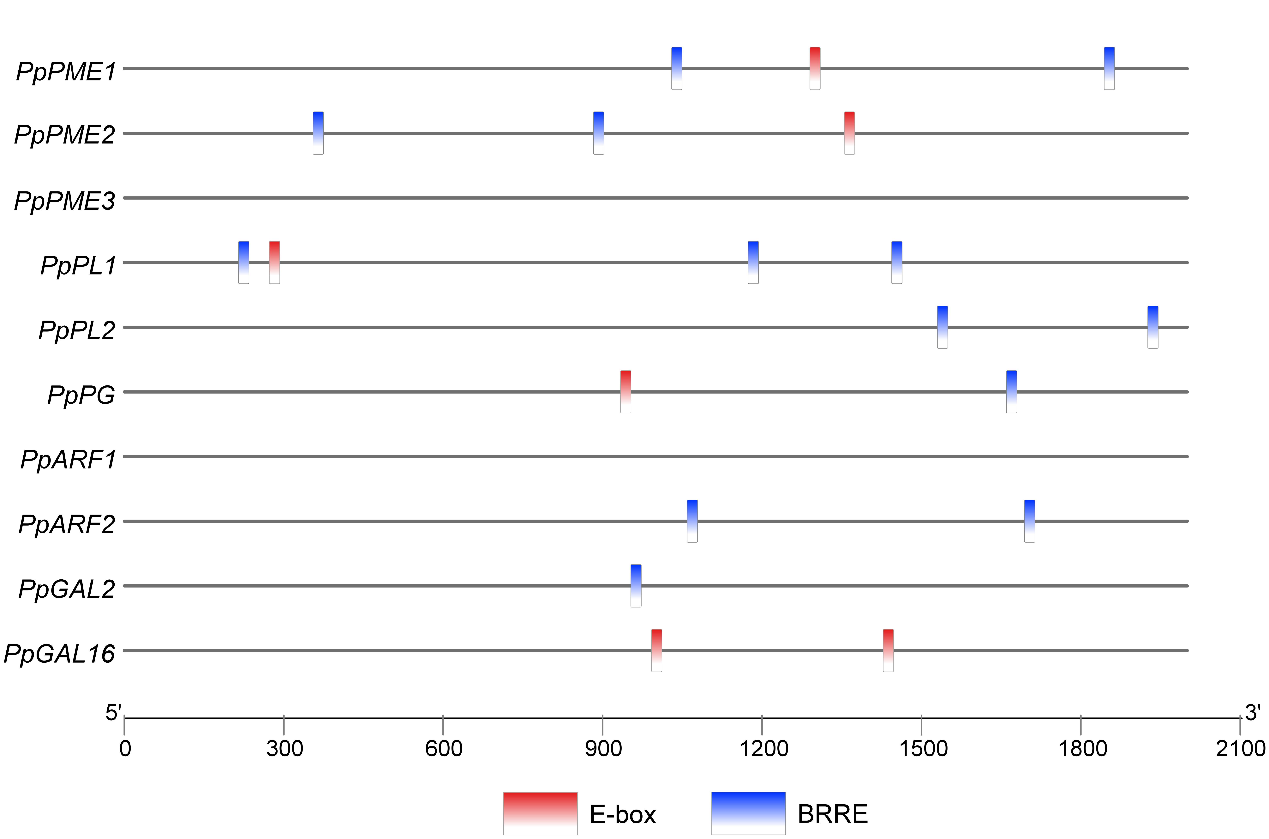


Figure. 2 Cis-element analysis of pectin degradation promoters.
